# Effect of Sulforaphane on Bladder Compliance in a Rat Model of Partial Bladder Outlet Obstruction

**DOI:** 10.1155/2019/6026719

**Published:** 2019-06-18

**Authors:** Chong Liu, Xiang Wan, Meng Gu, Yanbo Chen, Zhikang Cai, Juan Zhou, Qi Chen, Zhong Wang

**Affiliations:** Department of Urology, Shanghai Ninth People's Hospital, Shanghai Jiao Tong University School of Medicine, Shanghai, China

## Abstract

**Aims:**

To investigate the effect of Nrf2 activator sulforaphane (SFN) on bladder compliance and the underlying mechanisms in a rat model of partial bladder outlet obstruction (BOO).

**Methods:**

Male 8-week-old Sprague-Dawley rats were divided into three groups. BOO rats were given daily 0.5 mg/kg sulforaphane (BOO+SFN) or vehicle (BOO) intraperitoneally for 4 weeks, while sham-operated rats were treated with vehicle (Sham). Bladder compliance, histological alteration, and collagen deposition were evaluated. The expression levels of collagen I, collagen III, MMP-1, and TIMP-1 were measured by immunohistochemistry and western blotting.

**Results:**

BOO led to a significant decrease in bladder compliance. The change was partially restored by SFN treatment. The expression of MMP-1 was significantly decreased accompanying with increased TIMP-1 expression in BOO rats compared with that in Sham rats, which was ameliorated by SFN treatment. Moreover, the increased collagen I/collagen III ratio in the BOO group was reversed by SFN treatment.

**Conclusions:**

Sulforaphane suppressed collagen deposition by regulating the MMP-1 and TIMP-1 expression and decreasing the collagen I/III expression ratio in BOO rats and improved bladder compliance.

## 1. Introduction

Benign prostatic hyperplasia (BPH) is a common disease accompanied by lower urinary tract symptoms (LUTS) in older men [1]. More than 50% of men aged 50 years or older experienced some degree of bladder outlet obstruction (BOO) secondary to BPH, which has a significant impact on the patients' quality of life [2]. BOO mostly led to the decrease of bladder compliance, which has been known to be correlated with deterioration of renal function. From a biomechanical standpoint, physiological stretch increased the expression of extracellular matrix (ECM) proteins [3, 4]. Compliance is primarily related to extracellular matrix deposition. Increased deposition of extracellular matrix in the detrusor layer is the primary reason for decreased compliance. As in other organs [5], ECM deposition is dependent on the balanced activity of proteolytic enzymes, including matrix metalloproteinases (MMPs) and their endogenous inhibitors, tissue inhibitors of metalloproteinases (TIMPs) in the bladder. The imbalance between MMPs and TIMPs is a key regulator in ECM deposition [6].

Yang et al. [7] showed that the imbalance between MMP-1 and TIMP-1 favoured accumulation of ECM and associated with decreased bladder compliance in a rabbit BOO model. As the collagen components are mainly collagen types I and III in the bladder, collagen type I plays a vital role in the tensile resistance; however, the characteristic of collagen type III is strong expansibility [8]. Up to now, the relationship between bladder compliance and the expression of collagen type I and collagen type III in a BOO rat model remains unknown.

Increasing evidence has shown that ischemia and reperfusion are a major etiologic factor in the progression of bladder dysfunction induced by BOO and that part of the damage is because of the generation of reactive oxygen species (ROS) [9]. Our previous research [10] showed that sulforaphane (SFN), a Nrf2 agonist and antioxidant, could have a protective effect on bladder function by attenuating oxidative stress of the rat after BOO. SFN is a naturally occurring isothiocyanate which has been studied for its antioxidative and anti-inflammatory properties. However, it is still unclear whether sulforaphane improves bladder compliance and the underlying mechanisms remain to be elucidated.

We hypothesized that sulforaphane might have a beneficial effect on bladder compliance in BOO rats. The present study was performed to investigate the effect of sulforaphane on bladder compliance and collagen subtype and correlated them with MMP-1 and TIMP-1 expressions in the bladder of BOO rats.

## 2. Materials and Methods

### 2.1. Animals

8-week-old male Sprague-Dawley rats were used. Rats were housed by two per cage in a temperature-controlled room. Food pellets and tap water were supplied freely. A total of 18 rats were randomly divided into three groups: (1) sham-operated rats; (2) BOO rats; and (3) BOO rats treated with sulforaphane (0.5 mg/kg/day) intraperitoneally for 4 weeks. Sulforaphane was provided by Cayman Chemical (USA). Sulforaphane treatment was initiated immediately following the operation of BOO rats. The dose of 0.5 mg/kg/day SFN in this research has been proved effective in other researches. All experimental procedures were approved by the Animal Research Ethics Committee of Shanghai Jiao Tong University School of Medicine.

### 2.2. BOO Model

The bladder outlet was partially obstructed by the retropubic method described previously [11, 12]. Briefly, rats were anesthetized with 10% chloral hydrate and then placed in a supine position. The abdominal cavity was opened by a midline incision to expose the urethrovesical junction. A proximal urethra was loosely tied with a 19-G needle using a 3-0 silk thread, and the needle was removed to produce partial BOO. The same operation was performed in sham-operated rats without tying the thread.

### 2.3. Cystometry

Cystometry was performed on conscious rats 4 weeks after surgery to evaluate the urodynamic parameters as previously described [13, 14]. Briefly, rats were anesthetized and an abdominal midline incision was made. A purse string suture was placed in the dome of the bladder, into which a PE-50 catheter was inserted and fixed. After confirming that there was no leak around the catheter, the catheter was delivered subcutaneously to the dorsum and stored in a skin pouch. The abdominal incision was then closed. A cystometric analysis was performed 3 days later. The PE-50 catheter was connected via a 3-way stopcock to a pressure transducer for recording intravesical pressure and to a syringe pump for infusing saline. Cystometry was performed through the infusion of warm saline (37°C) at a rate of 12 mL/h. The cystometric parameters such as micturition pressure, bladder capacity, and compliance were recorded to evaluate bladder function.

### 2.4. Histological Analysis

The bladders were harvested and fixed in 4% paraformaldehyde-phosphate buffer. Specimens were then embedded in paraffin and cut into 5 *μ*m sections, which were stained with hematoxylin and eosin (HE) for general morphology; Masson's trichrome staining and Sirius red staining for collagen subtype were performed in all groups. Histological analysis was evaluated blind by a pathologist.

### 2.5. Immunohistochemical Staining

Sections were stained to visualize the expression of MMP-1 and TIMP-1 in different groups. Briefly, the slides were placed in a cuvette with 100 mL 0.01-M citrate buffer pH 6.0 and heated in boiling water for 30 minutes. The slides were then incubated in 3% H_2_O_2_ for 10 minutes to block endogenous peroxidase activity and treated with 0.2% Triton X-100 for 10 min, washed in PBS for 5 min. The slides were blocked with 5% bovine serum for 30 minutes in a humidified chamber and then incubated with primary antibody overnight at 4°C. Then, the slides were incubated for 45 minutes at room temperature in a humidified chamber with a biotinylated secondary antibody. Finally, the slides were incubated with DAB for 5 minutes and counterstained with Mayer hemalum.

### 2.6. Western Blotting

Bladder tissue was homogenized in RIPA lysis buffer containing a protease inhibitor cocktail. Protein extracts were quantified by the bicinchoninic acid method, and 20 *μ*g of protein was loaded per well onto a 7.5% SDS-PAGE gel, then fractionated and transferred to a nitrocellulose membrane by semidry electrotransfer for 3 h at 100 V. After soaking in blocking buffer, membranes were incubated overnight at 4°C with primary antibodies. Membranes were incubated with horseradish peroxidase-linked secondary antibody and visualized with a chemiluminescent detection system. Band intensity was quantified by densitometry. The values were normalized to GAPDH, and these results were expressed as fold change.

### 2.7. Statistical Analysis

Data were expressed as the mean ± S.E.M. One-way analysis of variance was used to analyze the statistical significance. *P* < 0.05 was considered to indicate a statistically significant difference.

## 3. Results

### 3.1. Cystometric Findings

**Figure 1:**
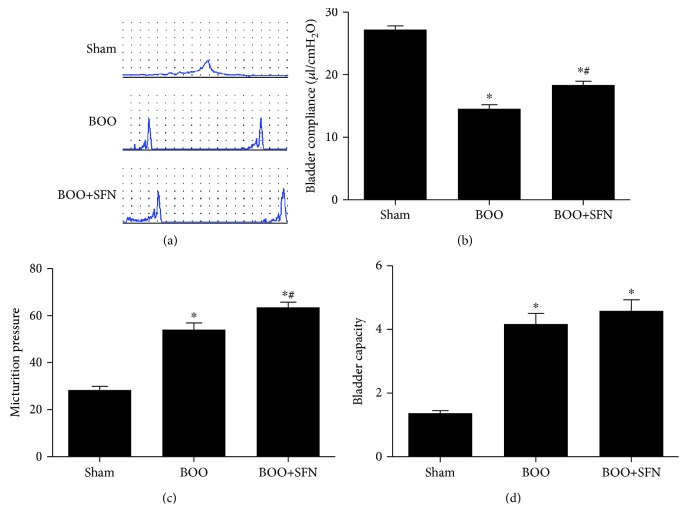
The effect of sulforaphane on urodynamic parameters in a rat model of BOO. Urodynamic curves (a), bladder compliance (b), micturition pressure (c), and bladder capacity (d). Cystometry was monitored 4 weeks after BOO surgery in conscious rats as described in Materials and Methods. Each bar represents the mean ± S.E.M.^∗^*P* < 0.05 compared with Sham; ^#^*P* < 0.05 compared with BOO.

Cystometry was performed on conscious rats 4 weeks after surgery. As shown in Figure 1(a), the urodynamic curves were recorded in different groups. Bladder compliance was 27.22 ± 0.60, 14.56 ± 0.66, and 18.36 ± 0.59 *μ*l/cmH_2_O in the Sham group, BOO group, and BOO+SFN group, respectively (Figure 1(b)). Bladder compliance was significantly lower in the BOO group than that in the Sham group; however, the bladder compliance was increased in the BOO+SFN group compared with that in the BOO group. Furthermore, micturition pressure was 28.40 ± 1.49, 54.0 ± 2.92, and 63.50 ± 2.22 cmH_2_O in the Sham group, BOO group, and BOO+SFN group, respectively (Figure 1(c)). Micturition pressure was significantly higher in the BOO+SFN group than that in the BOO group. Bladder capacity was 1.37 ± 0.08, 4.17 ± 0.33, and 4.58 ± 0.35 ml in the Sham group, BOO group, and BOO+SFN group, respectively (Figure 1(d)). Bladder capacity was significantly increased induced by BOO; however, no significant difference was observed in bladder capacity between the BOO and BOO+SFN groups.

### 3.2. Histological Analysis

**Figure 2:**
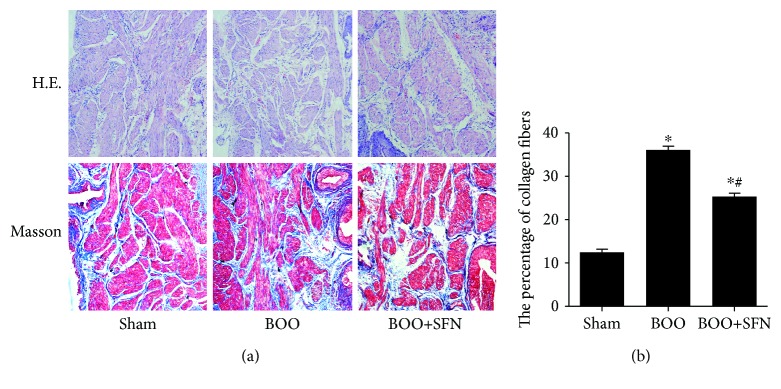
The effect of sulforaphane on bladder histology in BOO rats. A rat bladder was stained with hematoxylin-eosin (HE) and Masson's trichrome staining (Masson) (magnification ×100) (a) and the percentage of collagen fibers (b). Each bar represents the mean ± S.E.M.^∗^*P* < 0.05 compared with Sham; ^#^*P* < 0.05 compared with BOO.

**Figure 3:**
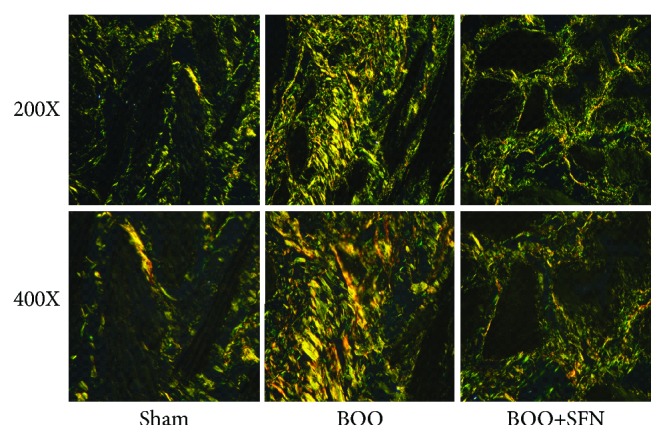
The effect of sulforaphane on the expression of collagen subtype in BOO rats. A rat bladder was stained with Sirius red staining (magnifications ×200 and ×400). Collagen type I was dyed yellow, and collagen type III was dyed green.

HE staining demonstrated that BOO caused obvious damage of the bladder structure. The space between smooth muscle bundles was evident, and the fracture of muscle bundles can be observed in the bladder of BOO rats. SFN treatment could attenuate these histological changes induced by BOO (Figure 2(a)). Based on the Masson's trichrome staining (Figure 2(a)), the area ratio of collagen fibers (type I/type III) was 12.37 ± 0.72, 36.03 ± 0.88, and 25.24 ± 0.91 in the Sham group, BOO group, and BOO+SFN group, respectively (Figure 2(b)). The deposition of collagen in the extracellular matrix significantly increased in BOO rats compared with that in sham-operated rats. The deposition of collagen was decreased in BOO+SFN rats compared with that in BOO rats. Furthermore, Sirius red staining was performed to measure the expression of collagen subtype in all rats (Figure 3). The result showed that the expression of collagen types I and III significantly increased in BOO rats compared to that in sham-operated rats. However, SFN treatment in BOO rats decreased the expression of collagen types I and III compared to that in BOO rats.

### 3.3. Immunohistochemical Analysis

**Figure 4:**
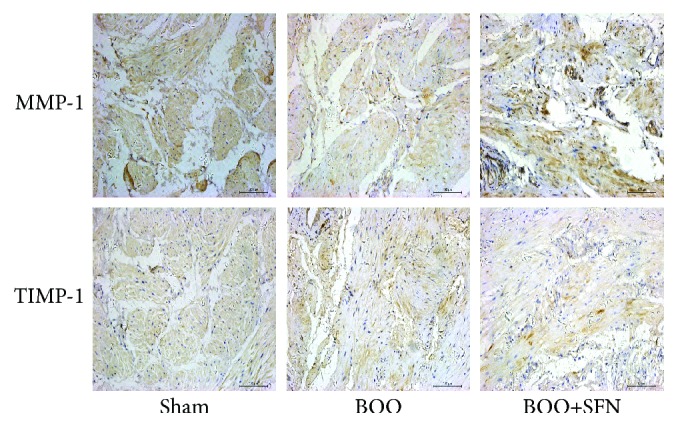
The effect of sulforaphane on the expression of MMP-1 and TIMP-1 in BOO rats. MMP-1 and TIMP-1 were evaluated by immunohistochemical staining (magnification ×200). The palm yellow represents positive expression in the bladder.

As shown in Figure 4, there was a significant decrease in abundance of MMP-1 staining in the bladder of BOO rats based on the immunohistochemical evaluation. SFN treatment in BOO rats significantly increased MMP-1 staining compared to that in BOO rats. Moreover, the TIMP-1 staining significantly increased in the bladder of BOO rats compared to that of Sham rats and SFN treatment ameliorated the high expression of TIMP-1 induced by BOO.

### 3.4. Western Blotting Analysis

**Figure 5:**
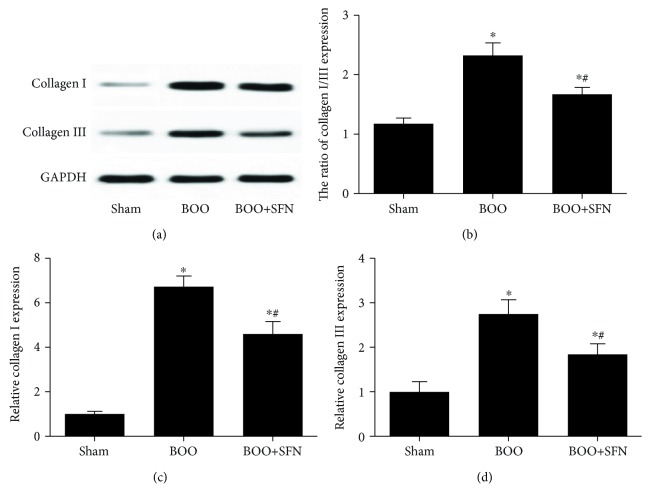
Western blotting analysis showing the expression of collagen types I and III in Sham, BOO, and BOO+SFN rat bladders. Collagen types I and III expression (a), the ratio of collagen types I/III (b), relative collagen I expression (c), and relative collagen III expression (d). Each bar represents the mean ± S.E.M.^∗^*n* = 3, *P* < 0.05 compared with Sham; ^#^*n* = 3, *P* < 0.05 compared with BOO.

**Figure 6:**
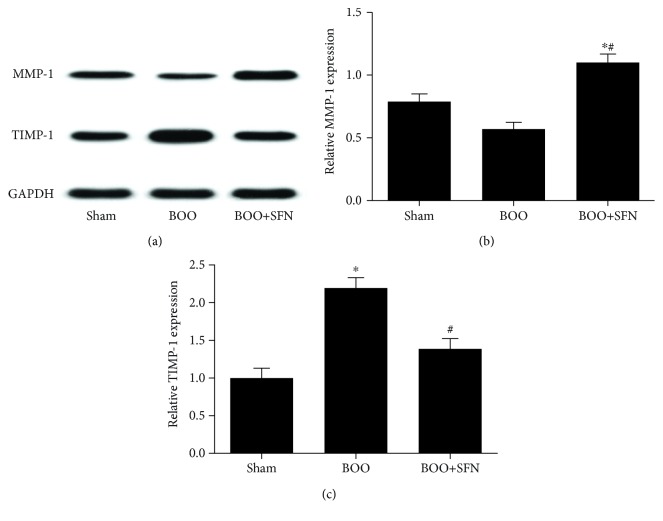
Western blotting analysis showing the expression of MMP-1 and TIMP-1 in Sham, BOO, and BOO+SFN rat bladders. MMP-1 expression was reduced while TIMP-1 expression was greater after BOO (a); semiquantitative data of MMP-1 based on western blot result (b) and semiquantitative data of TIMP-1 based on western blot result (c). Each bar represents the mean ± S.E.M.^∗^*n* = 3, *P* < 0.05 compared with Sham; ^#^*n* = 3, *P* < 0.05 compared with BOO.

The expression of collagen types I and III was significantly increased in BOO rats compared with that in Sham rats. SFN treatment in the BOO rats decreased collagen type I and III protein levels compared to that in the rats of the BOO group (Figure 5(a)). Furthermore, the ratio of collagen types I/III was 1.2, 2.3, and 1.7 in the Sham group, BOO group, and BOO+SFN group, respectively (Figure 5(b)). The ratio was higher in the BOO group compared to that in the Sham group. However, SFN treatment in BOO rats decreased the ratio of collagen types I/III compared to that in BOO rats. Figures 5(c) and 5(d) show that the expressions of collagen types I and III were both decreased in the BOO+SFN group compared to those in the BOO group. The expression of MMP-1 was significantly decreased in the BOO group compared with that in the Sham group; however, SFN treatment in BOO rats increased the expression of MMP-1 compared with that in BOO rats (Figures 6(a) and 6(b)). Furthermore, the TIMP-1 expression was significantly increased in BOO rats compared with that in Sham rats. The expression of TIMP-1 had some extent of decrease in BOO+SFN rats compared with that in BOO rats (Figures 6(a) and 6(c)). These results indicated that BOO mainly increased the expression of TIMP-1 in the bladder tissue, thus resulting in collagen deposition, while SFN treatment alleviated collagen deposition by increasing the expression of MMP-1 in BOO rats.

## 4. Discussion

BOO is known to induce morphological and functional changes of the bladder [15, 16]. Hypoxia and oxidative stress has been shown to be one of the pathophysiological factors in the bladder remodeling [17, 18]. In our previous research, SFN has been shown to have a protective effect on bladder function by attenuating oxidative stress in a BOO model [10]. However, whether SFN improves bladder compliance and the underlying mechanisms remains to be clarified. In the present study, we demonstrated the effect of Nrf2 activator SFN on bladder compliance and the underlying mechanisms in a rat model of BOO.

In order to reflect the real situation, bladder function was evaluated by cystometry in conscious rats. Our results revealed that the micturition pressure in the BOO+SFN group is higher than that in the BOO group. Moreover, SFN treatment improved bladder compliance in BOO rats.

We found that SFN treatment relieved the increased collagen deposition in the bladder of BOO rats. SFN treatment in the BOO rats increased the expression of MMP-1, while the level of TIMP-1 levels had some extent of decrease compared with BOO rats, which indicated that SFN could improve bladder compliance by regulating the expression of MMP-1 and TIMP-1 in BOO rats.

It has been reported that BOO induced the increased expression of collagen type I and III proteins in the muscular layer [19, 20]. Whether SFN has a role in regulating the expression of collagen types I and III in the BOO model has not been reported. In the study, the expression level of collagens I and III were downregulated in the BOO+SFN group.

To our knowledge, this is the first study to confirm the role of sulforaphane on bladder compliance associated with the expression of MMP-1/TIMP-1 and collagens I/III in a BOO rat model. We found that SFN protected against bladder dysfunction in rats with BOO. SFN treatment suppressed BOO-induced decreased bladder compliance by regulating the expression of MMP-1/TIMP-1 and collagens I/III in the bladder tissue.

There is some limitation in extrapolating our results to the real human situation. The protective effect of SFN may not be solely attributable to bladder compliance; it also plays a vital role in antioxidative and anti-inflammatory stress. Therefore, further studies are needed for a full understanding of the effect of SFN.

## 5. Conclusions

SFN treatment improved bladder compliance by upregulating the expression of MMP-1 and downregulating TIMP-1 expression. The decreased ratio of collagens I/III may also play an important role in improving bladder compliance in BOO rats.

## Data Availability

The data used to support the findings of this study are included within the article.
